# Construction and Application of the Financial Early-Warning Model Based on the BP Neural Network

**DOI:** 10.1155/2022/5108677

**Published:** 2022-10-13

**Authors:** Weiwei Jiang, Xuefeng Wu, Xi Wang

**Affiliations:** ^1^Applied Technology College, Soochow University, Suzhou 215325, Jiangsu Province, China; ^2^Business College, Soochow University, Suzhou 215021, Jiangsu Province, China; ^3^Economic College, Jiaxing University, Jiaxing 314001, China

## Abstract

In order to further improve the early-warning effect of enterprise financial crisis management and reduce the occurrence of enterprise financial crisis, by taking listed companies as examples and combining the operating conditions of listed companies, a financial crisis early-warning indicator system was built from five aspects of profitability, debt-paying ability, development ability, operation ability, and cash flow ability. In addition, a financial management early-warning model based on the BP neural network algorithm was built. Through the experimental prediction, it is showed that the financial crisis early-warning model of listed companies based on the BP neural network algorithm for crisis prediction accuracy was more than 75%. The accuracy of the first three years of model prediction was 93.33% and 72.34%, respectively. The accuracy of model prediction in the first two years was 94.67% and 82.98%, respectively. In the first year, the accuracy rate increased to 100% and 89.36%. Compared with the prediction accuracy of the logistic model (50%), it is fully reflected that the financial early-warning model proposed in the research had a good crisis prediction ability.

## 1. Introduction

At present, with the rapid development of China's market economic system reform, the Chinese enterprises are in the important stage of the internal deepening market economic system reform and the external further global economic integration. In this process, many contradictions are evident in small- and medium-sized enterprises and even large enterprises. Among them, enterprises are faced with increasingly high financial risks in the process of operation. In the environment of fierce market competition inside and outside, how to avoid financial risks through effective ways is the key issue that modern enterprises should consider [1]. The financial crisis early-warning management system is an important part of enterprise risk management. In the research, combined with the demand of financial risk management and with the aid of the BP neural network algorithm fusion, more suitable enterprise financial risk management and early-warning mechanism were explored [2]. Through the model experiment, the feasibility and accuracy of risk prediction of the early-warning model were verified.

**Figure 1:**
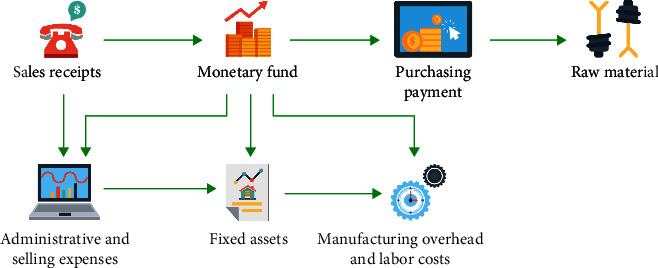
Financial early-warning model.

The research on the financial crisis early warning originated in the west. Since the 1930s, the constant emergence of the economic crisis caused a large number of economic losses in the Western developed countries. So, the government and scholars began to realize the need to forecast the economic crisis, thus reducing the economic loss. After that, a large number of scholars began to study the financial crisis of the enterprise [3]. In the early stage of the research, scholars mainly focused on the qualitative research, such as flow chart research method, management evaluation method, and four-stage analysis method. However, the qualitative research relied too much on the subjective thinking of researchers. The same method led to different conclusions, and the accuracy of early-warning was also very low. Therefore, scholars gradually began to use quantitative research methods, such as univariate analysis, multivariate analysis, logistic regression, and neural network analysis [4]. With the development of research, many researchers found that the accuracy of the univariate discrimination model was not high in practical application and there were great limitations. Therefore, scholars began to introduce the multivariate linear discriminant analysis method into the field of crisis warning. Some scholars studied the multivariate discriminant model. Firstly, five optimal indicators were extracted from 22 financial ratios, and then, the multivariate discriminant model was established to predict financial warning, as shown in Figure 1. Later, the method was innovated and the *Z*-value multivariable financial crisis warning model was established, which achieved great success. Based on the analysis of the relationship between the traditional financial crisis and income, a multivariate linear model was constructed and the accuracy and scientificity of the model was verified. Based on the establishment of the *Z* model, the listed companies of Chinese automobile manufacturing business were studied. And the research showed that the model had high accuracy [5]. Foreign scholars used the neural network prediction model. The results showed that the neural network prediction model was more effective than the discriminant analysis model. With the continuous development of computer technology, the application of the neural network early-warning model began to be popularized. Research studies showed that the artificial neural network had high efficiency and certain effectiveness in predicting bankruptcy risk [6]. The BP neural network model was used to investigate listed companies, and the results showed that the BP neural network model had an efficient predictive effect [7]. The BP neural network model was established to carry out early-warning research on Chinese listed companies, and it was found that the BP neural network model had high accuracy for financial early warning of listed companies [8]. In addition, models such as SVM vector machine, MDS multidimensional scale model, and genetic algorithm were also used for financial crisis warning of listed companies; each model had its advantages and disadvantages [9]. The univariate warning analysis method was simple to use and easy to use, but the accuracy of prediction was low, so it was gradually replaced by some other models. In general, the multiple linear regression model, multiple logistic regression model, and neural network model were popular in the field of early warning. Most studies showed that the BP neural network was superior to other models in crisis warning ability and had strong operability.

## 2. BP Neural Network Warning Model

### 2.1. Perceptron

Perceptron is the origin of the neural network, and it receives multiple input signals, multiplies them by a fixed weight, and adds bias to get the signal sum. Then, compared with threshold, when the signal sum is greater than the threshold, 1 is output, known as the “neurons are activated.” When signals sum is less than or equal to the threshold value, 0 is output, namely, neurons are not activated [10].

(1) $$\begin{array}{c} y=\left\{\begin{array}{c} \begin{array}{cc} 0, & w_{1}x_{1}+w_{2}x_{2}+b\leq \theta , \end{array}\\ \begin{array}{cc} 1, & w_{1}x_{1}+w_{2}x_{2}+b>\theta \text{.} \end{array} \end{array}\right. \end{array}$$

**Figure 2:**
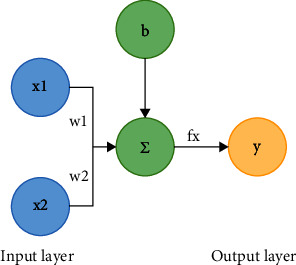
A single-layer perceptron with two inputs.

As shown in Figure 2, *x*_1_ an d *x*_2_ are the input data. *w*_1_ an d *w*_2_are the weight parameter. *b* is the bias, and *θ* is the threshold. Formula (1) is the activation function *fx*. The structure of single-layer perceptron is composed of these parts. Through this model, the linear problem can be solved. For example, without considering bias and activation function formula, we have

(2) $$\begin{array}{c} y=w_{1}x_{1}+w_{2}x_{2}\text{.} \end{array}$$

The above formula contains two parameters. All parameters can be found if there are two different samples. Assume that there are two sample data, as shown in Table 1.

Formula (3) can be obtained from Table 1.

(3) $$\begin{array}{c} \left\{\begin{array}{c} w_{1}+2w_{2}=5,\\ 5w_{1}+3w_{2}=11\text{.} \end{array}\right. \end{array}$$

The final solution is as follows:

(4) $$\begin{array}{c} \left\{\begin{array}{c} w_{1}=1,\\ w_{2}=2\text{.} \end{array}\right. \end{array}$$

Therefore, the parameter solution of the perceptron is dependent on the number of samples. The more nodes there are, the more connections between the nodes will be, the more the parameters will be, and the required sample data will also increase [11]. The significance of activation function is to make the output data meet the requirements and avoid too scattered output data after weight vector multiplication. Common activation functions are the sigmoid and ReLU functions.

#### 2.1.1. Sigmoid Function

**Figure 3:**
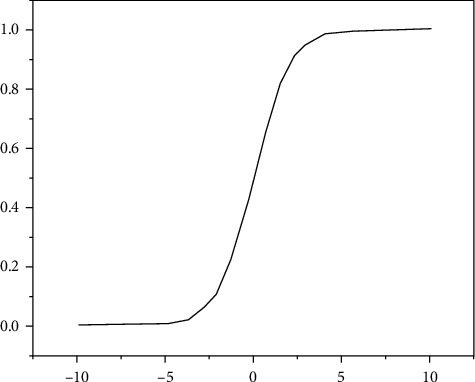
Sigmoid function image.

Sigmoid is the most widely used activation function, whose domain is (−∞, +∞) and range is (0, 1). The function is continuously smooth and differentiable everywhere in the domain. Because the range is (0, 1), the result is usually used as a probability. In the case of classification, generally, if the output after sigmoid function is greater than 0.5, the instance is regarded as classified into this category. And if it is less than or equal to 0.5, it is not classified into this category [12]. The function image is shown in Figure 3 and the formula is shown as follows:

(5) $$\begin{array}{c} sigmoid\left(x\right)=\frac{1}{1+e^{-x}}\text{.} \end{array}$$

#### 2.1.2. ReLU Function

The function formula is shown as follows:

(6) $$\begin{array}{c} ReLU\left(x\right)=\left\{\begin{array}{c} \begin{array}{cc} x, & x\geq 0, \end{array}\\ \begin{array}{cc} 0, & x<0\text{.} \end{array} \end{array}\right. \end{array}$$

**Figure 4:**
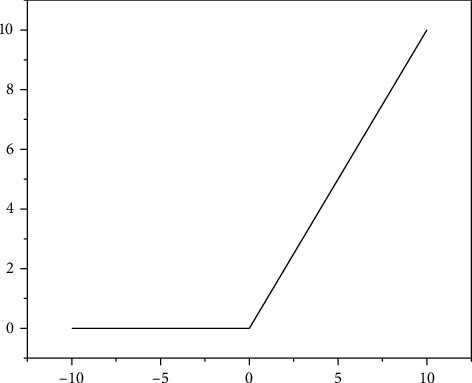
ReLu function image.

The ReLU function is a piecewise function that turns all negative values to 0 while positive values remain the same. In deep neural networks, ReLU function is mostly used as the activation function, because it is conducive to model convergence, as shown in Figure 4.

### 2.2. Neural Network

**Figure 5:**
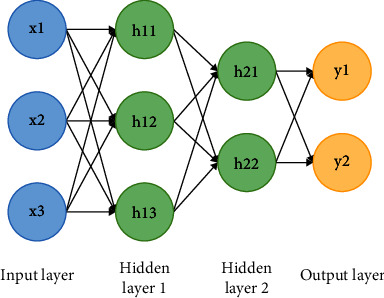
Neural network with two hidden layers.

In Figure 5, there are three neurons in the input layer, which means that the input vector is a three-dimensional vector. The signal of the input layer is multiplied by the weight matrix to obtain the signal sum, which becomes the input vector of the first hidden layer after the activation function operation. The first hidden layer has three neurons. These three neurons receive signals from the input layer. Multiplied by the weight matrix of this layer, it becomes the input vector of the second hidden layer through activation function operation. And the last layer is the output layer, with two neurons, which means the output vector is a two-dimensional vector [13]. This process of computation from front to back is called forward propagation. The characteristics of input vector are abstracted gradually through layer-by-layer operation, and finally, the classification effect is realized. There are 9 connections between hidden layer 1 and the front layer, corresponding 9 parameters (not considering bias), there are 6 connections between hidden layer 2 and the front layer (6 parameters), and there are 4 connections between the output layer and the front layer (4 parameters), altogether 19 parameters [14]. Because of the hidden layer, the multilayer neural network can simulate almost all nonlinear functions.

The commonly used error functions are mean square error function and cross entropy error function.

#### 2.2.1. Mean Square Error

The square of the difference between the elements of the corresponding correctly supervised data (label) of each output of the neural network is calculated, as shown in

(7) $$\begin{array}{c} E=\frac{1}{2}\underset{k}{\sum }{\left(y_{k}-t_{k}\right)}^{2}\text{.} \end{array}$$

In the above formula, *y*_*k*_ represents the output of the neural network. *t*_*k*_ represents the correct classification label. *k* represents the dimension of data [15].

#### 2.2.2. The Cross Entropy Error

Entropy is a measure of the amount of information contained in an event. The information entropy of an event is defined as

(8) $$\begin{array}{c} H\left(x\right)=-\overset{n}{\underset{i=1}{\sum }}p\left(x_{i}\right)\log\left(p\left(x_{i}\right)\right)\text{.} \end{array}$$

Cross entropy is used to measure the difference between two probability distributions, as shown in

(9) $$\begin{array}{c} H\left(p,q\right)=-\overset{n}{\underset{i=1}{\sum }}p\left(x_{i}\right)\log\left(q\left(x_{i}\right)\right)\text{.} \end{array}$$

*p* an d *q* represent two distributions, and *p*(*x*_*i*_)and *q*(*x*_*i*_) represent the probability of an event *x*_*i*_ occurring in one of these distributions, respectively. In addition, the log is the natural log. The larger the cross entropy is, the greater the difference between the two distributions is. So, it can be used as the loss function of the neural network model output and the difference between correctly classified labels [16].

The BP algorithm updates the weight matrix of the previous layer along the gradient direction that makes the error of the latter layer smaller and finally propagates it to the back layer to achieve the purpose of seeking the optimal weight matrix. The vector summarized by partial derivatives of all variables is called gradient. Suppose a function contains two variables, as shown in

(10) $$\begin{array}{c} f\left(x_{0},x_{1}\right)=x_{0}^{2}+x_{1}^{2}\text{.} \end{array}$$

Then, the vector ((*∂f*/*∂x*_0_), (*∂f*/*∂x*_1_)) is the gradient of the function *f*, and the following formula can be calculated:

(11) $$\begin{array}{c} \left(\frac{\partial f}{\partial x_{0}},\frac{\partial f}{\partial x_{1}}\right)=\left(2x_{0},2x_{1}\right)\text{.} \end{array}$$

The ultimate goal of the neural network is to find the parameter vector that minimizes the loss function. When the number of parameters is large, it is difficult to find the optimal solution directly. With the gradient method, the value of the function advances some distance from the current position along the gradient direction. Then, the gradient in the new direction is re-calculated. Then, it advances along the new gradient direction, and the function value is reduced again [17]. The mathematical formula of gradient in formula (11) is shown in

(12) $$\begin{array}{c} x_{0}=x_{0}-\eta \frac{\partial f}{\partial x_{0}}x_{1}=x_{1}-\eta \frac{\partial f}{\partial x_{1}}\text{.} \end{array}$$

The BP algorithm mainly includes three parts, namely, forward propagation, backpropagation, and weight updating (parameter updating). On the premise that the neural network transmits signals by means of the propagation mode and the connections between neurons do not cross layers, suppose a total of *N* + 1 layer networks, which are represented by Layer 0 to Layer *N*. The state of the *k* layer for a feature *p* is expressed as *x*_*p*_(*k*), and the state of the whole network for the feature *p* is expressed as *x*_*p*_. So, *x*_*p*_(0) is the input vector *I*_*p*_ and *x*_*p*_(*N*) is the output vector. The size of the output vector is the same as the sample label *D*_*p*_. Suppose there are *P* features in total, then the whole network (*p*=1...*P*) is composed of the states *X* of all features *x*_*p*_. *k* − 1 layer is connected with *k* layer through parameter vector W(*k*); then, the input value *A*_*p*_(*k*) of *k* layer is shown in

(13) $$\begin{array}{c} A_{p}\left(k\right)=W\left(k\right)X_{p}\left(k-1\right)\text{.} \end{array}$$

The formula for forward propagation can be expressed as follows:

(14) $$\begin{array}{c} x_{p}\left(k\right)=F\left(W\left(k\right)X_{p}\left(k-1\right)\right)=F_{k}\left(A_{p}\left(k\right)\right),\forall k\in \left[1,N\right]\text{.} \end{array}$$

In formula (14), *f*_*k*_represents the activation function of the *k* layer.

When the mean square error is used as the loss function, the Lagrange function of a single feature is shown as follows:

(15) $$\begin{array}{c} L_{p}\left(W,X_{P},B_{P}\right)={\left(D_{p}-X_{p}\left(N\right)\right)}^{T}\left(D_{p}-X_{p}\left(N\right)\right)\\ +\sum_{k=1}^{N}B_{P}{\left(k\right)}^{T}X_{p}\left(k\right)-F\left(W\left(k\right)X_{p}\left(k-1\right)\right)\text{.} \end{array}$$

The above formula is composed of two terms. The first term is the square of the difference between the output of the model *X*_*p*_(*N*) and the real label of the sample *D*_*p*_. The second conditional term is the sum of *N* sub-terms, each of which represents a layer, and each subterm is the dot product of a Lagrange multiplier vector *B*(*k*) and the state of the layer. When all conditions are met, all conditions are 0 and the following formula can be obtained:

(16) $$\begin{array}{c} X\left(k\right)=F\left[W\left(k\right)X_{p}\left(k-1\right)\right],\forall k\in \left[1,N\right]\text{.} \end{array}$$

The formula for finding the parameter solution that makes the function reach the minimum value is as follows:

(17) $$\begin{array}{c} \Delta L=\left(W,X,B\right)=0\text{.} \end{array}$$

The formula can be decomposed into three subformulas, as follows:

(18) $$\begin{array}{c} \frac{\Delta L\left(W,X,B\right)}{\partial B}=0, \end{array}$$

(19) $$\begin{array}{c} \frac{\Delta L\left(W,X,B\right)}{\partial X}=0, \end{array}$$

(20) $$\begin{array}{c} \frac{\Delta L\left(W,X,B\right)}{\partial W}=0. \end{array}$$

From layer *N* to layer *N* − 1 and then to layer *N* − 2, gradually reverse calculation to layer 1, so as to update the parameters of each layer, so that the overall error of the model becomes smaller, which is the principle of backpropagation. The optimization conditions that need to be followed when calculating parameter vector *W* can be decomposed into *N* seed types, as shown in

(21) $$\begin{array}{c} \frac{\Delta L\left(W,X,B\right)}{\partial W\left(k\right)}=0,\forall k\in \left[1,N\right]. \end{array}$$

The formula for updating parameter vectors using the gradient descent method is shown as follows (*λ* is step size):

(22) $$\begin{array}{c} W\left(k\right)\leftarrow W\left(k\right)-\lambda \frac{\Delta L\left(W,X,B\right)}{\partial W\left(k\right)}. \end{array}$$

That is, the classical parameter updating formula of the BP algorithm is as follows:

(23) $$\begin{array}{c} W\left(k\right)\leftarrow W\left(k\right)+\lambda \sum_{p=1}^{P}Y_{p}\left(x\right)X_{p}^{T}\left(k-1\right). \end{array}$$

In the specific training of the BP neural network, firstly, the number of nodes of input layer, hidden layer, and output layer should be set, respectively. The initial value of the training sample, weight between nodes, and output threshold should be marked at the same time. Second, after making all the preliminary preparations, the training sample data is input through the input layer, and the errors between the calculation results of the hidden layer and the output layer are analyzed during the conduction process. The weights and thresholds of different nodes are adjusted and finally the error function model is obtained. If the error is small, the results can be directly output. If the error is large, it will reverse conduction to the second step, and the loop will continue until the data output is completed.

## 3. Establishment of the Financial Risk Early-Warning Model for Listed Companies

### 3.1. Sample Selection

In the research, 374 listed pharmaceutical companies in the WIND database were selected for research. As shown in Table 2, according to the financial status of the sample companies from 2013 to 2021, the sample was divided into three types, namely, financial health, financial risk, and major financial risk, including 231 financial health enterprises, 102 financial risk enterprises, and 41 major financial risk enterprises. Meanwhile, the total sample was divided into two categories. The first was the training sample of the training neural network model, consisting of 249 enterprises, including 155 financial health enterprises, 67 financial risk enterprises, and 27 major financial risk enterprises. The other is the testing sample, a total of 125 enterprises, including 76 financial health enterprises, 35 financial risk enterprises, and 14 major financial risk enterprises.

After removing a small amount of abnormal or missing data, 3312 groups of panel data were retained. The data in the research came from WIND and CSMAR databases and were finally processed and analyzed by SPSS18.0 and MATLAB2017b software.

### 3.2. Screening of Early-Warning Indicators Based on Industry Characteristics

**Figure 6:**
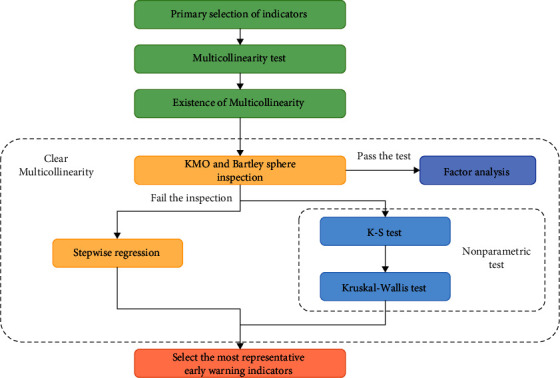
Screening ideas of early-warning indicators.

The screening ideas of early-warning indicators in the research are shown in Figure 6 and the process is shown as follows.

The first step is the primary selection of indicators. Based on the commonly used financial risk early-warning indicators and the characteristics of the pharmaceutical industry, there are a total of 21 variables selected in the primary selection of early-warning indicators.

The second step is to perform the multicollinearity test on the primary indicators. Because the number of 21 variables in the primary selection is large and may be correlated with each other, the multicollinearity of the indicator needs to be tested. From the test results, it is found that the 21 variables in the primary selection fail to pass the multicollinearity test.

The third step is to eliminate the multicollinearity of the primary indicator system. First of all, the failure of KMO test indicates that the indicator system of primary selection is not suitable for common factor analysis methods. Therefore, the research further screens the indicators of primary selection by combining the stepwise regression method and the nonparametric test method. Finally, the most representative 8 early-warning indicators are obtained.

Considering the industry characteristics of listed pharmaceutical companies in China and the difficulty of data acquisition, 21 basic indicators are initially selected to reflect the characteristics of sample companies. There are a total of 21 different variables in the primary indicators in the research, with a large number of variables overall. From the definition of the indicator, there is a high possibility of correlation between the two. Therefore, it is necessary to determine whether multicollinearity exists by calculating the correlation coefficient of the primary indicator system. The multicollinearity correlation statistics of the primary indicator system are shown in Table 3.

According to the correlation statistics of the multicollinearity test, among the 21 indicators in the primary selection, the allowance of 10 indicators is less than 0.1 and the coefficient of variance expansion of 13 indicators is greater than 5. Therefore, multicollinearity exists among the 21 indicators in the primary selection, which requires further screening of indicators. There are many methods to eliminate multicollinearity in the indicator system, and factor analysis is generally considered first. However, KMO and Bartlett sphericity tests are carried out on the primary indicator system in the research, and it is found that the primary indicator system fails the test and is not suitable for factor analysis. The corresponding relationship between the suitability of factor analysis and KMO statistics is shown in Table 4.

The test results show that most of the KMO values of the primary indicator system in the research are in the range of 0.5∼0.7, indicating that the primary indicator system is not suitable for factor analysis. If factor analysis is forced to reduce data dimension, it will lead to a lot of information loss and may directly affect the accuracy of the warning model. Therefore, in the case of factor analysis being unsuitable, the method of stepwise regression is used to analyze and screen the indicator system of the primary selection. In the research, stepwise regression method was only used to screen the primary indicator system in the first step, and the screening standard should not be too strict. Therefore, the *F*-test threshold of the eliminated variables was set as 0.2, and the significance level of the introduced variables was set as 0.15. The model summary of the final stepwise regression equation is shown in Table 5. It can be seen from Table 5 that the finally determined stepwise regression model is Model 13, and the *R*-square value is 0.656, indicating that the combination of indicators in Model 13 can explain 65.6% of the dependent variables and has a high goodness of fit. Therefore, a total of 13 variables were screened from the primary indicator system with a significance level of 0.15 by the stepwise regression method in the research.

When identifying whether the distribution of a variable is significantly different among multiple samples, the sample normality test is usually carried out first. If the sample as a whole is normally distributed, the *t*-test can be used directly. If the sample as a whole does not follow a normal distribution, nonparametric tests are usually used. First of all, the *K*-*S* test is used to test the normality of all warning indicators of *t* − 1, *t* − 2, and *t* − 3 phases of the sample population. As can be seen from Table 6, most of the financial indicators in each period do not follow the normal distribution at the significance level of 0.05. Therefore, the *t*-test method cannot be used for indicator screening, and the nonparametric test method should be used for further screening.

Nonparametric tests are usually used to compare whether the results of different treatments are consistent in different distributions. The *K*-*W* test adopted in the research can effectively identify whether the sample population is consistent in different samples with random distribution. The test results are shown in Table 7. A total of 8 indicators are different at the significance level of 0.05 in *t* − 3 phase, 9 indicators are different at the significance level of 0.05 in *t* − 2 phase, and 13 indicators are different at the significance level of 0.05 in *t* − 1 phase.

In summary, analysis and screening are carried out in combination with stepwise regression and *K*-*W* suggestions. Finally, eight representative indicators are determined as *X*1 rate of return per share, *X*2 operating profit rate, *X*4 net profit growth rate, *X*5 main business income growth rate, *X*7 total assets growth rate, *X*9 inventory turnover rate, *X*17 debt guarantee rate, and *X*20 management expense rate, respectively, reflecting the company's profitability, cash flow capacity, growth capacity, and nonfinancial indicators, four aspects of information.

### 3.3. Design of the BP Neural Network Model

**Figure 7:**
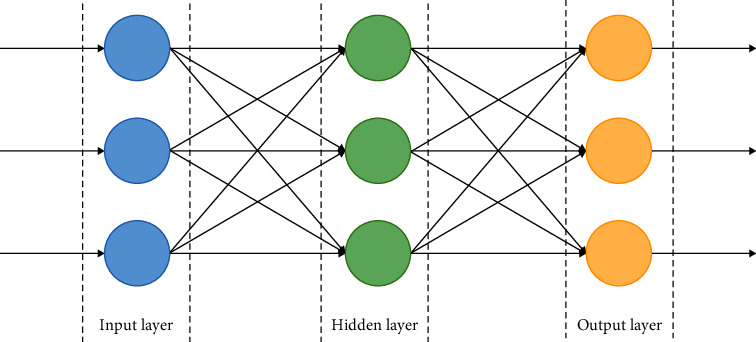
Three-layer BP network structure.

Taking the three-layer BP neural network as an example, as shown in Figure 7, all nodes between each two layers are interconnected. In fact, hidden layers can have one or more layers, but the most common and practical is still a three-tier structure.

**Figure 8:**
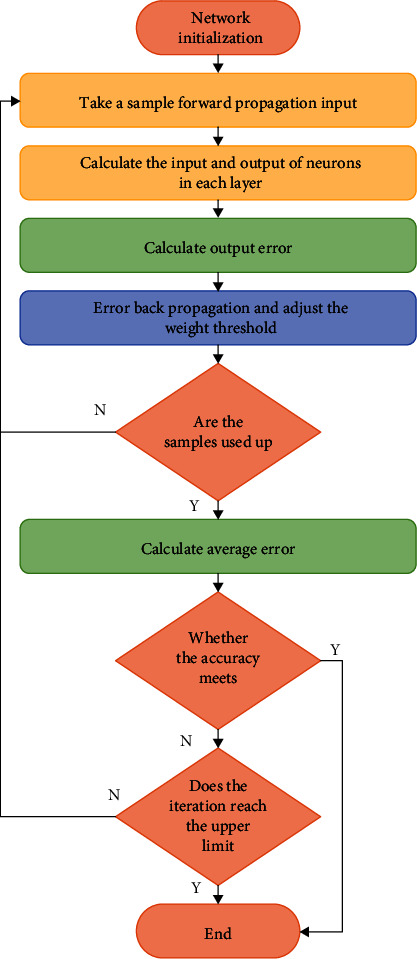
The procedure flow of the BP algorithm.

First, the number of nodes of the input layer, hidden layer, and output layer should be set, respectively. In addition, the initial value of the training sample, weight between nodes, and output threshold should be marked. Second, after making all preliminary preparations, the training sample data is input through the input layer. The errors between the calculated results of the hidden layer and the output layer are analyzed during the conduction process. The weights and thresholds of different nodes are adjusted to obtain the error function model. Whether the error value deviates from the range is analyzed. The calculation can be completed if there is no deviation. Otherwise, the data should be resubstituted into the second step for repeated calculation until the error value is within the allowed range. The procedure flow of the BP algorithm is shown in Figure 8.

### 3.4. Analysis of Prediction Results of the BP Neural Network Model

Matlab2017b software was used to input 8 financial indicator sample data of 245 training samples. After the neural network ran 10 times, the training error reached the predetermined precision. The correlation between the predicted value and the actual value of the test sample enterprise was relatively high, and the *R* value reached 0.91766. The test samples were input into the trained BP neural network model for testing. Part of the test results is shown in Table 8 (3 decimal places are reserved).

After summarizing the test results in Table 8, the test results of the enterprise financial early-warning model are shown in Table 9.

From the annual data test, in the first three years, before the outbreak of financial risk, model prediction accuracy was 93.33% and 72.37%, respectively. The accuracy of model prediction in the first two years was 94.65% and 82.98%, respectively. In the first year, the accuracy rate increased to 100% and 89.37%, respectively. The closer the outbreak of financial risk is, the more accurate the model judgment will be. From the overall perspective of the test results, the model designed in the research will help to build a more practical financial risk early-warning system for Chinese pharmaceutical listed companies. Compared with other models, the model designed in the research has higher prediction accuracy and is easier to operate. What is particularly important is that in practical application, enterprise employees have uneven grasp of information technology, so the simple and stable financial risk warning model is more practical.

### 3.5. Specific Case Analysis

**Figure 9:**
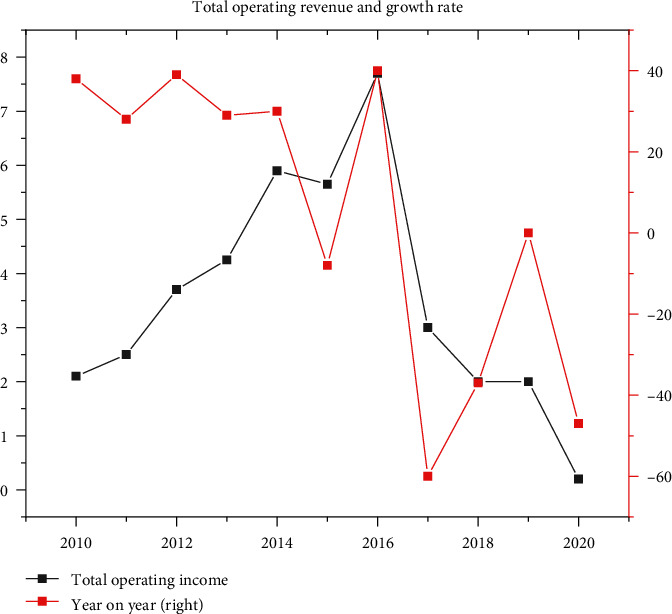
Change of *S* Company's total operating income from 2013 to 2021.

*S* Company was taken as an example. As shown in Figure 9, in terms of the company's business scale and total income, the trend of *S* Company was relatively stable before it started to expand aggressively in 2017, showing a steady upward trend as a whole. Until 2018, it suddenly showed a downward trend. Although it picked up again the following year, it was due to the high nonoperating income brought by the performance betting agreement in the M&A process. Since 2020, the business scale and total income of S Company began to decline rapidly year by year.

**Figure 10:**
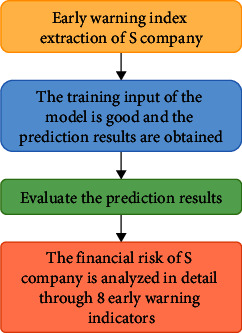
Application simulation flow of the *S* Company model.

Taking S Company as an example, this section mainly simulates how to make use of the early-warning model proposed in the research to predict the actual application of the enterprise. The specific process is given in Figure 10.

The 8 early-warning indicators of S Company from 2018 to 2020 are shown in Table 10. Among them, 8 early-warning indicators are *X*1 rate of return per share, *X*2 operating profit rate, *X*4 net profit growth rate, *X*5 main business income growth rate, *X*7 total assets growth rate, *X*9 inventory turnover rate, *X*17 debt guarantee rate, and *X*20 management expense rate, respectively, reflecting the company's profitability, cash flow capacity, growth capacity, and operating capacity, four aspects of information.

As can be seen from Table 11, when the result is (1, 0, 0), the financial health of the next year is predicted. When the result is (0, 1, 0), the financial risk for the next year is predicted. When the result is (0, 0, 1), the financial risk for the year after that is predicted. In 2018, three years before the outbreak of financial risks, the early-warning model predicted that *S* Company had financial risks. As time goes by, in 2019 and 2020, the model predicted that *S* Company would have significant financial risks.

The main reason for the decline of *S* Company's main business income is the dispersion of its main business. In the process of rapid development and expansion, *S* Company once extends its main business to several popular sectors but fails to achieve good results. After the main business keeps changing, its traditional business always occupies the majority. The gross profit rate of products accounts for more than 10% of the operating revenue of *S* Company from 2019 to 2021, as shown in Table 12. As can be seen from Table 12, the gross profit rate of its main business products in 2020 is greatly reduced. However, the increase in 2021 is due to the sharp drop of production cost in the upstream of the supply chain, which directly leads to the increase of its gross profit rate.

Except for 2014, the debt guarantee rate of *S* company reached the peak in 2015 and reached the bottom in 2020. In other years, although relatively stable, the rate remained low. As can be seen from the above analysis, the reason for the high debt guarantee rate in 2015 was that the cash received by the company from selling goods and providing services increased in that year and the company did not conduct debt financing on a large scale, so the debt guarantee rate was high. From 2018 to 2020, *S* Company conducted debt financing on a large scale, making its total debt increase rapidly. Although the net cash flow generated by operating activities was relatively high in 2018, the debt guarantee rate did not decrease, but it began to decline in 2019 and reached the bottom when the temporary payment of 1 billion yuan was withdrawn without cause in 2020. From 2013 to 2019, the rate of management expense remained relatively stable as the management expense and main business income of *S* Company kept increasing at the same time. Due to the sharp decline of main business income after 2019, *S* Company's management expense rate also began to rise rapidly after 2019. To sum up, the early-warning model designed in the research based on the BP neural network model could stably and effectively predict the financial risks of Chinese pharmaceutical listed companies. Taking *S* Company as an example, the hidden financial risks could be predicted three years before the outbreak of financial risks only through 8 indicator data of three years. If financial risks could be prevented or controlled effectively in time, the economic losses caused by the outbreak of financial risks may be reduced and even eliminated.

## 4. Conclusions

Based on the financial performance of 374 listed pharmaceutical companies from 2013 to 2021, the sample was divided into three types, namely, financial health, financial risk, and major financial risk. Considering the financial characteristics of listed pharmaceutical companies in China and the difficulty of obtaining data, 21 early-warning indicators were selected. Through multiple screening, an early-warning indicator system composed of 8 variables was constructed. Finally, the BP neural network model was designed and trained to give a stable and accurate financial risk warning for Chinese pharmaceutical listed companies. Four indicators reflecting profitability and two indicators reflecting cash flow capacity had significant differences in each phase, indicating that profitability indicators and advanced flow indicators could be more stable and effective in predicting the financial risks of Chinese pharmaceutical listed companies. However, the two indicators of operating capacity had significant differences only in the latest phrase, indicating that operating capacity indicators had strong short-term forecasting ability. In the first three years of the outbreak of financial risks, as time approached, the number of indicators with significant differences between the sample companies with financial health also increased, indicating that financial risks may expand from a single indicator to many indicators as time went by. From the annual data inspection, three years before the outbreak of financial risk, model prediction accuracy was 93.33% and 72.34%. The accuracy of model prediction in the first two years was 94.67% and 82.98%, respectively. In the first year, the accuracy rate increased to 100% and 89.36%. The closer the outbreak of financial risk is, the more accurate the model judgment would be. The final results showed that the BP neural network model designed in the research could predict the financial risks of Chinese pharmaceutical listed companies stably and effectively. At the same time, the accuracy of prediction also proved the feasibility of the early-warning indicator system. Therefore, the analysis of early-warning indicators could also investigate the cause of financial risk scientifically and effectively.

## Figures and Tables

**Table 1:** Sample data of single-layer perceptron.

Sample number	*x*1	*x*2	*y*
1	1	2	5
1	4	4	11

**Table 2:** Sample data classification.

	Training set	Testing set	Total
Financial health	155	76	231
Financial risk	67	35	102
Major financial risk	27	14	41
Total	249	125	374

**Table 3:** Multicollinearity correlation statistics.

Variable	Nonstandardized coefficient		Standardized coefficient		Significance	Colinear statistics	
	B	Standard error	Beta	T		Allowance	VIF
(Constant)	1.336	0.455		2.938	0.0005		
*X*1	−0.461	0.235	−0.365	−1.953	0.056	0.107	9.301
*X*2	−0.744	0.483	−0.403	−1.546	0.128	0.056	18.577
*X*3	−2.600	2.223	−0.286	−1.167	0.248	0.063	15.503
*X*4	1.201	0.667	1.092	1.775	0.082	0.011	100.335
*X*5	−0.122	0.154	−0.086	−0.841	0.405	0.382	2.647
*X*6	0.000	0.002	−0.023	−0.263	0.799	0.584	1.712
*X*7	−0.073	0.138	−0.043	−0.543	0.599	0.601	1.676
*X*8	0.002	0.002	0.078	0.916	0.365	0.537	1.888
*X*9	−0.002	0.014	−0.031	−0.145	0.876	0.090	11.291
*X*10	−0.301	0.284	−0.133	−1.066	0.298	0.240	4.142
*X*11	0.343	0.233	0.211	1.479	0.151	0.188	5.406
*X*12	0.311	0.263	0.543	1.120	0.245	0.019	59.943
*X*13	−0.133	0.264	−0.220	−0.503	0.621	0.020	49.615
*X*14	−0.294	0.747	−0.088	−0.398	0.699	0.089	11.452
*X*15	0.111	0.102	0.333	1.054	0.299	0.041	24.833
*X*16	0.087	0.192	0.055	0.472	0.644	0.251	4.066
*X*17	−0.114	0.123	−0.721	−0.951	0.351	0.007	157.601
*X*18	0.023	0.098	0.099	0.248	0.802	0.023	42.153
*X*19	−0.776	0.369	−0.156	−2.101	0.043	0.726	1.401
*X*20	0.995	1.044	0.255	0.941	0.351	0.054	18.977
*X*21	−2.454	2.705	−0.077	−0.921	0.369	0.498	2.006

**Table 4:** Test criteria for KMO statistics.

KMO	Factor analysis of fitness
KMO > 0.9	Very suitable
0.8 < KMO < 0.9	Relatively suitable
0.7 < KMO < 0.8	Suitable
0.6 < KMO < 0.7	Not suitable
0.5 < KMO < 0.6	Very poor
KMO < 0.5	Not suitable

**Table 5:** Model summary.

Model	*R* ordinary	*R* squared after adjustment	Errors in standard estimation
1	0.426	0.423	0.53389
2	0.525	0.522	0.48672
3	0.554	0.546	0.47431
4	0.578	0.566	0.46278
5	0.591	0.583	0.45521
6	0.607	0.594	0.44721
7	0.620	0.603	0.44161
8	0.631	0.621	0.43469
9	0.636	0.624	0.43456
10	0.641	0.623	0.43221
11	0.647	0.625	0.43091
12	0.652	0.629	0.42876
13	0.656	0.631	0.42676

**Table 6:** The normality test.

Indicator	*t* − 1		*t* − 2		*t* − 3	
	*Z*	*P*	*Z*	*P*	*Z*	*P*
*X*1	0.153	0.001	0.163	0.001	0.173	0.001
*X*2	0.243	0.001	0.305	0.001	0.253	0.001
*X*3	0.136	0.015	0.130	0.007	0.097	0.001
*X*4	0.322	0.001	0.301	0.001	0.243	0.001
*X*5	0.088	0.201	0.292	0.001	0.436	0.001
*X*6	0.402	0.001	0.456	0.001	0.165	0.001
*X*7	0.166	0.001	0.118	0.027	0.167	0.001
*X*8	0.415	0.001	0.414	0.001	0.371	0.001
*X*9	0.292	0.001	0.531	0.001	0.441	0.001
*X*10	0.173	0.001	0.136	0.004	0.178	0.003
*X*11	0.135	0.005	0.176	0.001	0.161	0.001
*X*12	0.179	0.001	0.187	0.001	0.179	0.001
*X*13	0.149	0.001	197.000	0.001	0.204	0.001
*X*14	0.065	0.202	0.076	0.202	0.076	0.202

**Table 7:** List of indicators with significant differences.

*t* − 1		*t* − 2		*t* − 3	
Index	Sig.	Index	Sig.	Index	Sig.
*X*1	0.000	*X*1	0.000	*X*1	0.000
*X*2	0.000	*X*2	0.000	*X*2	0.000
*X*3	0.000	*X*3	0.000	*X*3	0.000
*X*4	0.000	*X*4	0.000	*X*4	0.000
*X*5	0.000	*X*6	0.006	*X*5	0.001
*X*6	0.000	*X*7	0.000	*X*17	0.006
*X*7	0.000	*X*16	0.000	*X*18	0.007
*X*8	0.000	*X*17	0.000	*X*20	0.032
*X*9	0.039	*X*20	0.000		
*X*10	0.012				
*X*16	0.006				
*X*17	0.003				
*X*18	0.000				
*X*20	0.005				

**Table 8:** Partial test results.

	Expected result			Test result (*t* − 1)			Test result (*t* − 2)			Test result (*t* − 3)		
*A*	1	0	0	0.997	0	0	1	0	0	0.498	0.487	0.026
*B*	1	0	0	1	0	0	1	0	0	0.39	0.601	0.036
*C*	1	0	0	1	0	0	1	0.975	0	0.810	0.408	0.092
*D*	1	0	0	1	0	0	1	0	0	0.233	0.821	0.037
*E*	0	1	0	0.002	0.990	0	0	1	0	0.026	0.993	0.01
*F*	0	1	0	1	0	0	0.983	0	0	0.153	0.995	0.017
*G*	0	1	0	0	1	0	0	1	0.032	0.014	0.46	0.595
*H*	0	0	1	0	0	1	0	0	1	0	0.018	0.998
*I*	0	0	1	0	0	1	0	0	1	0	0.179	0.983
……	…	…	…	…	…	…	…	…	…	…	…	…

**Table 9:** Test results of the financial risk early-warning model.

Sample type	*t* − 1 phase		*t* − 2 phase		*t* − 3 phase	
	Financial health	Financial risk	Financial health	Financial risk	Financial health	Financial risk
Number of samples	75	47	75	47	75	47
Number of mistakes	0	5	4	8	5	14
Accuracy of judgment (%)	100	89.37	94.65	82.98	93.33	72.37

**Table 10:** Financial risk early-warning indicators of *S* Company.

Indicators	Year	2018	2019	2020
*X*1	Rate of return per share	0.04	0.05	−0.21
*X*2	Operating profit rate	0.12	0.11	−1.26
*X*4	Net profit growth rate	0.52	−0.13	−5.44
*X*5	Main business income growth rate	0.53	0.22	−0.71
*X*7	Total assets growth rate	0.88	0.36	0.04
*X*9	Inventory turnover rate (times)	0.23	0.34	0.12
*X*17	Debt guarantee rate (%)	0.07	−0.03	−0.38
*X*20	Management expense rate	0.22	0.20	0.44

**Table 11:** Financial risk early-warning simulation results of S Company from 2018 to 2020.

Year	Actual output value			Predicted results
	O_1_	O_2_	O_3_	
2020	0	0	1	(0, 0, 1)
2019	0.001	0	0.999	(0, 0, 1)
2018	0.012	0.942	0.143	(0, 1, 0)

**Table 12:** *S* Company accounts for more than 10% of the operating income of the product gross rate.

Product	2019	2020	2021
Plastic bottle large infusion production automatic line	83.44	28.61	46.55
Pharmaceutical packaging material	24.55	19.97	20.53

## Data Availability

The datasets used during the current study are available from the corresponding author on reasonable request.
